# A critical appraisal of epidemiological studies comes from basic knowledge: a reader's guide to assess potential for biases

**DOI:** 10.1186/1749-7922-2-7

**Published:** 2007-03-15

**Authors:** Stefania Boccia, Giuseppe La Torre, Roberto Persiani, Domenico D'Ugo, Cornelia M van Duijn, Gualtiero Ricciardi

**Affiliations:** 1 Institute of Hygiene, Catholic University of the Sacred Heart, Rome, Italy; 2 Department of Surgery, Catholic University of the Sacred Heart, Rome, Italy; 3 Department of Epidemiology & Biostatistics, Erasmus Medical Center, Rotterdam, The Netherlands

## Abstract

Scientific literature may be biased because of the internal validity of studies being compromised by different forms of measurement error, and/or because of the selective reporting of positive and 'statistically significant' results. While the first source of bias might be prevented, and in some cases corrected to a degree, the second represents a pervasive problem afflicting the medical literature; a situation that can only be 'corrected' by a change in the mindset of authors, reviewers, and editors. This review focuses on the concepts of confounding, selection bias and information bias, utilising explanatory examples and simple rules to recognise and, when possible, to correct for them. Confounding is a mixing of effects resulting from an imbalance of some of the causes of disease across the compared groups. It can be prevented by randomization and restriction, and controlled by stratification, standardization or by using multivariable techniques. Selection bias stems from an absence of comparability among the groups being studied, while information bias arises from distorted information collection techniques. Publication bias of medical research results can invalidate evidence-based medicine, when a researcher attempting to collect all the published studies on a specific topic actually gathers only a proportion of them, usually the ones reporting 'positive' results. The selective publication of 'statistically significant' results represents a problem that researchers and readers have to be aware of in order to face the entire body of published medical evidence with a degree of scepticism.

## Background

Epidemiology is largely a non-experimental discipline and although this has often been perceived as a weakness, probably its most important strength is in that observations are made on samples of real-world populations. In fact, the limited opportunity for experiments in epidemiological research has lead to a very critical theoretical understanding of the different types and sources of error [1].

Clinicians and surgeons face two important questions as they read medical research: is the report believable, and, if so, is it relevant to my practice? Uncritical acceptance of published research has led to serious errors and squandered resources [2,3]. In this review, we will examine these questions in terms of study validity; describing a simple checklist for readers to judge reported associations.

Additionally, we will describe another kind of bias, one that does not affect the validity of a single study but rather the dissemination of research findings: publication bias, which can be described as the selective submission or publication of study results based on the direction or strength of the study's findings.

### Modern definition of bias in epidemiology

Important discussions on bias took place as the concept of study design in modern epidemiology was refined. In the 1950s, the introduction of the randomised controlled trial (RCT) and its assigned role as a 'gold standard' for medical research lead to the anticipation that the validity of a study could be improved in circumstances where randomization was feasible. In the 1970s, during general discussions on the sources of biases, taxonomies of bias were proposed by Murphy and Sackett [4,5], with the latter proposing a 'catalogue' of 35 different types of bias. Modern definitions of bias, however, tend to be restricted to those categories that have a logical basis. According to Rothman, bias is a *systematic error *that afflicts study design, thus affecting the *validity *of the study itself [6]. An epidemiologic study can be viewed as an attempt to obtain an epidemiologic measure, with the 'correct' values unknown, therefore epidemiologists attempt to reduce this source of error through the careful design and analysis of the study itself.

A study can be biased due to an inability to completely control a confounding factor (*comparison bias*), the way in which the subjects have been selected (*selection bias*), or the way the study variables are measured (*information bias/misclassification*). A completely different source of error in epidemiological measures is *random error*, which affects the *precision *of a study, usually expressed by the use of confidence intervals (or ideally confidence interval functions). While this type of error is not discussed in this review it is important to note that random error is closely associated with sample size and it approaches zero as the study size increases, systematic errors on the other hand are not affected by study size.

### Confounding

Confounding can be defined as a bias in estimating an epidemiologic measure of effect resulting from an imbalance of other causes of disease in the compared groups [7]. It can also be defined as a mixing or blurring of effects, which comes about when a researcher attempts to relate an exposure to an outcome, but actually measures the effect of a third factor, termed a confounding variable [8]. Confounding is the most likely cause of a spurious association, which occurs when one factor, which is not in itself causally related to a disease, is unfortunately related to a range of other factors that do increase disease risk.

Before we proceed to defining a confounder, it would perhaps be easier to illustrate the concept with a simple example. Results from a large case-control study of IntraUterine Devices (IUDs) indicated a significant increase in salpingitis soon after insertion [9]. However, among married or cohabiting woman with only one reported sex partners in the past 6 months, no significant increase in risk was evident [10]. In the study, exposure to sexually transmitted diseases apparently confounded the association. Even among woman at lower risk for salpingitis, frequent coitus might increase risk of infection [11], and few studies have controlled for this variable.

So, for a characteristic to be a confounder in a particular study, it must simultaneously meet three criteria [7]:

1. it must be associated with the outcome in terms of prognosis or susceptibility

2. it must be associated with the exposure

3. it cannot be an intermediate cause, or in other words it must not be an effect of the exposure

We should however, keep in mind that not every predictor of disease occurrence is a confounding factor. For confounding to occur, a predictor of disease occurrence must also be in a state of imbalance across the exposure categories [7]. For example, let us suppose that age is a risk factor for a given disease, as it usually is. Then age would not be a confounder unless the age distribution of the people in the two exposure categories differed, which would result in the comparison of two different age groups. Under these circumstances, the effect of exposure will be confounded by the effects of age to an extent that would depend upon on the strength of the relationship between age and the disease, as well as the extent of the age imbalance across the exposure categories [7]. As such, information on the distribution of potential confounders with in the two comparison groups, is usually provided in the first table of a paper (Table 1) [12], where the most common strategy for identifying important imbalances for some of the covariates between the two groups is derived from the use of significance tests such as the χ^2 ^test (for dichotomous variable) or *t *test (for continuous variables), with their associated *p*-values, or the effect measure e.g. Odds Ratios, which is usually preferable, since it provides both the strength of an association and the precision of the estimate. However, we should keep in mind that in order for a characteristic to be considered as a confounder, it also needs to meet the two additional criteria previously mentioned.

**Table 1:** Baseline characteristics of head and neck cancer cases and controls according collected variables[12]

**Variable**	**Cases n (%)**	**Controls n (%)**	*p *value*
***Age groups (years)***			0.168
< 60	35 (28.2)	88 (35.3)	
> 60	89 (71.8)	161 (64.7)	
Total	124	249	
***Gender***			0.123
Male	105 (84.7)	194 (77.9)	
Female	19 (15.3)	55 (22.1)	
Total	124	249	
***Weight ± SD (Kg)***	72.70 ± 12.40	73.09 ± 12.15	0.775†
***Alcohol consumption***			0.001
No alcohol	37 (30.3)	107 (44.8)	
1–30 gr/die	53 (43.4)	122 (51)	
> 30 gr/die	32 (26.2)	10 (4.2)	
Total	122	239	
***Fruit intake***			0.197
≤ 1/day	69 (57.0)	153 (76.5)	
>1/day	52 (43.0)	86 (23.5)	
Total	121	239	
***Vegetable intake***			0.678
≤ 1/day	95 (78.5)	183 (76.6)	
>1/day	26 (21.5)	56 (23.4)	
Total	121	239	
***Smoking status***			0.001
Never smokers	17 (14.0)	133 (55.6)	
Ever smokers	104 (86.0)	106 (44.4)	
Total	121	239	
***Physical activity***			0.001
Never	109 (90.8)	172 (72.0)	
1–4 times/month	2 (1.7)	43 (18.0)	
>4 times/month	9 (7.5)	24 (10.0)	
Total	120	239	
***Solvents***			0.003
No	115 (95.0)	238 (99.6)	
Yes	6 (5.0)	1 (0.4)	
Total	121	239	
***Paints ***			0.004
No	114 (94.2)	238 (99.2)	
Yes	7 (5.8)	2 (0.8)	
Total	121	240	

* χ^2 ^test used to test statistically significant differences between groups; † Student's t-test

Although currently available evidence helps identify potential confounders, the imperfect state of knowledge means that some characteristics related to the outcome may not have been previously discovered (unknown confounders) [13]. In RCTs, all potential confounders (known and unknown) are expected to be evenly distributed between the groups being compared, thanks to the randomisation procedure. On the other hand, observational studies in epidemiology (mainly case-control and cohort-studies) are not protected in the same way and are especially vulnerable to unknown confounders. For example: in 1991 a meta-analysis investigating the association between the use of hormone replacement therapy (HRT) and the risk of coronary heart disease concluded that HRT halved the risk [RR= 0.50; 95% confidence interval (CI): 0.43–0.56] [14]. Results of a large RCT, however, were disappointing, showing no clear benefit (OR= 1.11; 95%CI: 0.96–1.30) [15], and women were left wondering what they should do. This illustrates that associations reported in observational studies but not confirmed in RCTs tend to be due to exposures that are related to socioeconomic and behavioural measures that are in turn related to disease (confounding).

Most probably, woman who use HRT are less likely to be smokers, more likely to exercise regularly, and less likely to come from lower socioeconomic classes, all of which reduce the risk of coronary heart disease. As Davey Smith and Ebrahim said, 'the inadequately recognised truth is that we live in associational world – people who are disadvantaged in one regard tend to be disadvantaged in other regards, since the forces that structure life chances and experience tend to ensure that some folk get the worst of all thing' [16].

#### What can we do about confounding?

Given the limited range of confounders measured in many studies and the inevitable stable degree of measurement error in assessing potential confounders, the standard statistical techniques poorly 'control' for confounding. It is however, worthwhile to describe the current methods being used to prevent and control for confounding. Beside the widespread concept of preventing confounding with the *randomization *(random assignment of subjects to experimental groups), which is only feasible in RCTs, confounding can also be efficiently prevented by the use of *restriction*.

Restriction represents the simplest approach: for example, if alcohol consumption is suspected to be a confounding factor in an association aiming to study the effect of tobacco smoking to laryngeal cancer, a study can enrol only non-drinkers. Restriction is a very efficient way to prevent confounding in any study, however often not used by epidemiologists who advocate that the representativeness of a study might be compromised by restriction. As Rothman states, this is indeed a fallacious way to think about the scientific inference, 'whose aim is to infer an abstract theory which is not tied to a specific population, and not to look for inferring a conclusion that would apply to a specific target population' [7].

Another way to prevent confounding is by *matching*, which is very common in case-control studies. Matching consists of selecting a comparison series that has an identical distribution to that of the index series for one or more covariates [7]. For example, in a case-control study where smoking is deemed a confounding factor, cases and controls can be matched by smoking status, so that for each case who smokes, a control who smokes is found. This approach, however, has at least three drawbacks: if matching is done for several potential confounders, the recruitment process might be difficult; by definition, it is impossible to examine the effect of a matched variable and lastly, which is less intuitive, in case-control studies matching can introduce confounding if the matching variable is highly correlated with the exposure [6]

When controlling for confounding, three of the methods can be employed: *stratification, standardization *and *multivariable modelling*. The first and last one are usually more commonly adopted. Stratification can be considered as a form of post-hoc restriction, done during the analysis. For example, results can be stratified by levels of the confounding factor. The Mantel-Haenszel (MH) procedure [17] combines the various strata into a summary statistics that describes the effect. If the MH adjusted effect differs substantially from the crude effect, then confounding is deemed present, and in these cases the adjusted estimate is the better estimate to use. Let's consider the fictitious example in Table 2, derived from Grimes DA [8]. The use of IUDs in this hypothetical cohort of 2,000 women is associated with the development of salpingitis (RR= 3.0; 95%CI: 1.7–5.4). However, when the data are stratified according to the number of partners, the resulting RR is 1.0 in each of the stratum, indicating no association between the IUD and salpingitis. The MH weighted RR, which controls for the confounding effect, is 1.0 (95%CI: 0.5–2.0), indicating that the apparent increase in risk was all due to confounding bias.

**Table 2:** Example of confounding in a hypothetical cohort study of intrauterine device use and salpingitis [8]

			Salpingitis			
			**Yes**	**No**	**Total**	**Proportion with salpingintis**
All women (n = 2,000)	Use of IUD	**Yes**	45	955	1,000	4·5 %
		**No**	15	985	1,000	1·5 %
		Crude RR=4.5%1.5%=3.0 (95% CI 1.7−5.4) MathType@MTEF@5@5@+=feaafiart1ev1aaatCvAUfKttLearuWrP9MDH5MBPbIqV92AaeXatLxBI9gBaebbnrfifHhDYfgasaacH8akY=wiFfYdH8Gipec8Eeeu0xXdbba9frFj0=OqFfea0dXdd9vqai=hGuQ8kuc9pgc9s8qqaq=dirpe0xb9q8qiLsFr0=vr0=vr0dc8meaabaqaciaacaGaaeqabaqabeGadaaakeaacqqGdbWqcqqGYbGCcqqG1bqDcqqGKbazcqqGLbqzcqqGGaaicqqGsbGucqqGsbGucqGH9aqpdaWcaaqaaiabisda0iabc6caUiabiwda1iabcwcaLaqaaiabigdaXiabc6caUiabiwda1iabcwcaLaaacqGH9aqpcqaIZaWmcqGGUaGlcqaIWaamcqqGGaaicqGGOaakcqaI5aqocqaI1aqncqGGLaqjcqqGGaaicqqGdbWqcqqGjbqscqqGGaaicqaIXaqmcqGGUaGlcqaI3aWncqGHsislcqaI1aqncqGGUaGlcqaI0aancqGGPaqkaaa@51FC@				

			Salpingitis			
			**Yes**	**No**	**Total**	**Proportion with salpingintis**

Women with 1 sexual partner (n = 1,200)	Use of IUD	**Yes**	3	297	300	1·0%
		**No**	9	891	900	1·0%
		RR=1.0%1.0%=1.0 MathType@MTEF@5@5@+=feaafiart1ev1aaatCvAUfKttLearuWrP9MDH5MBPbIqV92AaeXatLxBI9gBaebbnrfifHhDYfgasaacH8akY=wiFfYdH8Gipec8Eeeu0xXdbba9frFj0=OqFfea0dXdd9vqai=hGuQ8kuc9pgc9s8qqaq=dirpe0xb9q8qiLsFr0=vr0=vr0dc8meaabaqaciaacaGaaeqabaqabeGadaaakeaacqqGsbGucqqGsbGucqGH9aqpdaWcaaqaaiabigdaXiabc6caUiabicdaWiabcwcaLaqaaiabigdaXiabc6caUiabicdaWiabcwcaLaaacqGH9aqpcqaIXaqmcqGGUaGlcqaIWaamaaa@3B08@ (95% CI 0.27-3.67)				

			Salpingitis			
			**Yes**	**No**	**Total**	**Proportion with salpingintis**

Women with >1 sexual partner (n = 800)	Use of IUD	**Yes**	42	658	700	6·0 %
		**No**	6	94	100	6·0 %
		RR=6.0%6.0%=1.0 MathType@MTEF@5@5@+=feaafiart1ev1aaatCvAUfKttLearuWrP9MDH5MBPbIqV92AaeXatLxBI9gBaebbnrfifHhDYfgasaacH8akY=wiFfYdH8Gipec8Eeeu0xXdbba9frFj0=OqFfea0dXdd9vqai=hGuQ8kuc9pgc9s8qqaq=dirpe0xb9q8qiLsFr0=vr0=vr0dc8meaabaqaciaacaGaaeqabaqabeGadaaakeaacqqGsbGucqqGsbGucqGH9aqpdaWcaaqaaiabiAda2iabc6caUiabicdaWiabcwcaLaqaaiabiAda2iabc6caUiabicdaWiabcwcaLaaacqGH9aqpcqaIXaqmcqGGUaGlcqaIWaamaaa@3B1C@ (95% CI 0.43-2.29)				

In multivariable techniques, mathematical modelling examines the potential effect of one variable while simultaneously controlling for the effect of many others. Its major advantage is that it can control for more factors than stratification can, so that, for example, in a study investigating the association between oral contraceptive use and ovarian cancer risk, the investigator can simultaneously control for the effects of age, race, family history, parity, etc. One of the main drawbacks from the investigators viewpoint is the loss of hands-on feel for the data [8].

### Selection bias

'Selection biases are distortions that result from procedures used to select subjects and from factors that influence study participation' [7]. It usually occurs because the relationship between the exposure and the disease is different for those who do and those who theoretically could participate in the study, including those who do not take part. Selection bias can be related to the selection of cases (people with disease) or controls (people without the disease under study) in case-controls studies; the different probabilities of selecting people exposed or not exposed to a specific risk (or protective) factor in cohort-studies; due to a high proportion of individuals lost to follow-up or to a mistake in the analysis phase (e.g., do not adhere to the *intent-to-treat *principle) in prospective studies.

Because the association between exposure and disease among non-participants is usually unknown, the presence of selection bias is usually inferred, rather than observed [7]. In case-control studies, for example, if there is a different proportion of responders in the case series than in the control series, a selection bias might occur. If the proportion of responders is higher in the case series (e.g. 95%) and lower in the control ones (e.g. 70%), there would be the possibility that non-responders could have a different exposure history, and this could affect the real association measure between the exposure and the disease. A typical method used to recognise this is to compare responders and non-responders for a selection of variables that might be related to the main variable of interest. In a paper by De Vito et al. [18], aiming to evaluate the risk factors for obesity in children and adolescents, the authors calculated that a population of 2,053 students would need to be recruited, but only 1,357 students (66% of the sampled population) entered the study. They were unable to recruit the remaining 696 children as their parents did not provide consent, however the possible bias introduced by the high proportion of non-responders might be negated because, with respect to mean age, gender, and socio-economic factors, the non-responders were similar to the children who did enter the study.

A well-recognised form of selection bias comes from the self-selection of participants in some studies (*healthy volunteer effect*), or from the selection of healthier individuals by the investigator. In many studies relating to worker health the comparison of the death rate of workers with those of the general population (*healthy worker effect) *is biased because the general population contains many people that cannot work because of some form of illness.

#### Berkson's bias

A particular type of selection bias was described by Berkson in 1946, also known as admission-rate bias [19]. Many researchers prefer to select the control series within the hospital, instead of selecting them from the general population from which cases arise. According to Berkson, the relative prevalence of disease *x *in a group of patients who are hospitalised for disease *y *is inherently biased when compared with the population served by the hospital. In other words, this bias results from different rates of hospital admission for cases and controls. In fact, Berkson's argument applies in particular to hospital-based case-control studies in which one or more risk factors are studied in relation to the risk of a specific disease. Hospital rates depend on several circumstances, such as symptoms severity, ability to cure the disease and the reputation of a specific hospital regarding the treatment of a specific disease. Moreover, if a patient has more than one disease simultaneously, each disease could present with differing degrees of severity, with the direct consequence of this being a higher percentage of patients with multiple diagnoses within the hospital. It can be assumed that a hospitalised patient would have a higher likelihood of being exposed to a specific risk factor under study, with respect to the non-hospitalized population. Hence a discrepancy between hospitalised populations and source populations for the cases exists. If we would like to study the potential influence of a risk factor on a specific disease, then using controls derived from a hospital population could artificially increase the exposure rates amongst controls. In a case-control study using hospital controls, conducted by Sadetzki et al. [20], researchers investigated the role of several risk factors on the development of bladder cancer. Among these risk factor, smoking was associated with a non-significant 30% increased risk of developing bladder cancer (OR = 1.3; 95% CI: 0.8 – 2.5). Later Sadetzki et al. re-analysed the data, considering the possibility that a Berkson bias could have occurred in the previous analysis [21]. They removed patients with pulmonary disease from the control series, because the probability of these patients being exposed to tobacco smoking is higher than in the non-hospitalized population. After controlling for this possible source of bias, they found a significantly increased risk of bladder cancer for people exposed to tobacco (OR = 1.78; 95% CI: 1.05 – 2.99).

### Information bias

Information bias results from the incorrect determination of outcome and/or exposure, which can results in the *misclassification *of the exposure or the outcome of interest. There are two major types of misclassification bias [22]:

• *Non-differential misclassification bias: *when the misclassification is the same across the groups to be compared, in a manner that is not dependent on the exposure or disease state. For example, exposure is equally misclassified in cases and controls because a questionnaire is not properly designed to facilitate the collection of the level of the exposure, or when after the collection, some arbitrary cut-off value is used. For binary variables the estimate is biased toward the null value; however, for variables with more than two categories (polytomous) this rule may not hold and an away from the null bias can be obtained [23].

• *Differential misclassification bias: *when misclassification is different in the groups to be compared, usually occurring when the probability to properly detect an outcome depends on the exposure or disease state. The result is that the estimate is biased in either direction, toward the null or away from the null. Two common sub-categories of differential misclassification are detection bias and recall bias. The first one arises when a different way of measuring a variable among two groups is employed. If, as an example, in a case-control study, the cases are in-patients in a certain hospital and the researchers are interested in measuring blood pressure, we can expect an accurate measure, while in population controls the measurement could involve the self-use of blood pressure cuffs and consequently higher or lower levels of blood pressure may be detected. This type of bias can be corrected for after adjusting the recorded levels to that of a predetermined "normal" value [24].

In most cases, however, it is not possible to correct measurement bias (i.e., in measuring the height of people with their shoes on, in order to avoid measurement bias we must know the height of the shoe's heel, however, the easiest solution would be to measure the peoples height without shoes), therefore, efforts to avoid this type of misclassification should be taken in the design of the study. Sensitivity analyses, however, might help to quantify and possibly remove both selection and information bias [25]. A study conducted by La Torre at al. [26], carried out in Central Italian prisons, showed a prevalence of seropositivity of 26% for HBV, 28.2% for HCV and 5.4% for HIV. However, the data regarding the prevalence of these three viruses could represent a biased estimation of the phenomenon, as screening for these infections is not mandatory for all prisoners (HIV test in 47% of the total of prisoners). In this study it is possible that drug addicts were more likely to be given a HIV, HBV and HCV test (diagnostic suspicion bias) and as such prevalence rates may have been overestimated [8]. If a sensitivity analysis had been undertaken, based on the assumption that all prisoners not given a test were seronegative, then the authors would have found prevalence rates of 10.9%, 11.6%, and 2.45% respectively.

Recall bias is a differential misclassification bias that occurs in case-control studies, and eventuates when there is a difference in the accuracy and completeness of exposure information between cases and controls; as cases tend to think more about the possible factors affecting their disease status than controls, searching for all possible explanations for its development.

Thus, it is plausible that cases recall previous exposure to risk factors better than controls. For example, a recall bias could occur if the researchers are studying the possible association between some risk factors and congenital anomalies. In such cases, mothers with abnormal babies tend to cogitate deeper and have a higher motivation for searching their memories to identify possible explanations for the infant's disease. A particular situation can arise in case-control studies in which cases are deceased [27], and the researcher needs to select controls in a more appropriate way. In this case, 'next of kin' responders need to be selected for both cases and controls; the appropriate choice of controls would be to select dead controls. But, even if surrogate responders are involved, the investigator needs to know that the completeness and the detail of the answer depends on several factors, such as the type of kinsfolk (brothers tend to recall better childhood experiences, while husbands and wives answer questions related to adulthood better) or the gender of the responders [28]. Other ways of reducing recall bias can involve collecting information on past exposures obtained from sources independent of a person's memory (e.g. administrative databank), blinding interviews (i.e. without knowledge of disease status) and using standard questionnaires and measurement tools for exposure [29].

### Publication bias

Publication bias may be seen as a type of selection bias afflicting the scientific literature. It has long been recognised that only a proportion of research projects ultimately reach publication in an indexed journal [30]. Scherer et al showed that only about half of abstracts presented at conferences are later published in full. Similarly, 20% of trials funded by the National Institutes of Health remained unpublished several years after completion [31,32]. The fact that a substantial proportion of studies remain unpublished, even a decade after they have been completed and analysed is of concern, as potentially important information remains hidden from reviewers. Making the situation worse, is the issue that the dissemination of research findings is not a random process; rather it is strongly influenced by the nature and direction of the results.

For many years investigators have raised concerns that studies with 'negative' results may remain unpublished and their failure to appear in the literature can distort the conclusions that we obtain from clinical experiments regarding best practice [33]. In a 1979 article on "The 'file drawer' problem and tolerance for null results", Rosenthal described a gloomy scenario where "the journals are filled with the 5 per cent of the studies that show Type I errors, while the file drawers back at the lab are filled with the 95% of the studies that show non significant (e.g., *p *> 0.05) results" [34]. It is now well established that the probability of publication may be a function of the estimated intervention effect *θ *for whatever reason, with studies showing 'significant' results more likely to be published than those presenting 'negative' results [35], for example, in the field of emergency medicine, around 80% of the published literature shows 'statistically' significant results [36].

'Significant' results tend to be published in international journals, while 'non-significant results', when published, tend to appear in less renowned international journals or in the local literature, resulting in language bias (the "tower of Babel" bias) [37]. The opposite phenomenon, a reverse tower of Babel bias, nevertheless has also been described in which most of the locally produced and published literature is spuriously statistically significant [38]. However, ascertaining the extent of this type of bias is difficult, and typically we have no idea to what extent unpublished data distorts the literature. Retrieving unpublished data is currently very problematic, however unpublished results often represents 'negative' results from none-the-less well-conducted studies. Whittington et al [39] found that published data, on which clinical guidelines have been based, showed the drug fluoxetine to be advantageous is treating childhood depression, while additional unpublished studies reversed this result. The *Lancet *editorial [40] drew attention to the seriousness of these findings. In the last few years, however, many journals implemented dedicated sections to the publication of "null results", most often in the format brief papers. Even though this phenomenon appears admirable at a first sight, it confirms the connate vocation to dedicate more space to the reporting of positive studies, over studies that are equal in terms of power and quality [41].

Why do 'negative' results remain unpublished? The most reasonable explanations for this is that authors may fail to write them up and submit them to journals, as results such as these (from intervention or non-experimental studies) are reviewed less favourably, or because editors simply don't want to publish negative results. The peer review process is notoriously unreliable and susceptible to subjectivity, bias and conflict of interest [30].

In conclusion, a research environment that promotes and rewards only results that reach formal statistical significance is likely to foster data dredging and will create a distorted literature with very low credibility [41] and we need to be made aware of this in today's world efficacious medical care and evidence-based guidelines, which are after all meant to be based on the synthesized evidence from published studies.

## Conclusion

Here we have defined a simple checklist that can help identify potential confounders, as well as selection and information bias in published scientific literature. Furthermore, we have briefly shown that the concept of publication bias represents a problem that we all need to be concerned about, and in order to eliminate this issue there needs to be change in all of our mindsets.

## Abbreviations

OR = Odds Ratio; RR = Risk Ratio or Rate Ratio; CI = Confidence Interval

## Competing interests

The author(s) declare that they have no competing interests.

## Authors' contributions

Conception: GR

Drafting the manuscript: SB, GLT, RB

Critical revision: DDU, CMVD
